# 21.2 FAMILIAL RISK FOR SCHIZOPHRENIA

**DOI:** 10.1093/schbul/sby014.086

**Published:** 2018-04-01

**Authors:** David Braff

**Affiliations:** University of California, San Diego

## Abstract

**Background:**

Larry Seidman, Ph.D. was a key contributor to the Consortium on the Genetics of Schizophrenia (COGS) with its focus on understanding the genetic substrates of quantitative endophenotypes in schizophrenia patients. With his deep knowledge of neurocognition related to psychosis, Larry was able to help steer the COGS-1 family study of over 300 families. The subsequent COGS-2 case-control study used the same well curated, quality controlled extensive battery of testing with 1411 schizophrenia patients and 1500 extensively tested healthy control subjects. Larry was first author on the 2015 paper “Factor structure and heritability of endophenotypes and schizophrenia: findings from the Consortium on the Genetics of Schizophrenia.” It is important to note that related association studies examined the relationship of quantitative endophenotypes and genetic loci, and this is complementary to but distinct from case-control studies. These COGS studies identified a 42-gene network with a NRGL-ERBB4 hub underlying schizophrenia neurocognitive deficits. Thus, these Ns, modest for case-control studies, are quite powerful for gene finding using quantitative endophenotypic markers related to core “thought disorder” neurocognitive deficits in schizophrenia. These quantitative measures are up to 100 X more efficient and 10 X more powerful for gene finding than case-control studies as explained by Blangero, Williams and Almasy as early as 2005. Genes for SZ overlap with genes for key functionally important quantitative endophenotypes as shown by many groups, including COGENT and COGS, so case-control and endophenotype studies are best viewed as complementary in nature.

**Methods:**

Seidman et al (2015) examined 12 heritable neurocognitive and neurophysiological domains (including the Penn CNB Battery), as well as three neurophysiological measures reflecting inhibitory reprocessing from EEG and eye movements. Seidman et al’s analysis revealed five distinct factors in the composite COGS battery. These were 1-episodic memory, 2-working memory, 3-perceptual vigilance, 4-visual abstraction and 5-inhibitory processing. The five factors had similar structures across probands, siblings and controls. Also, heritability was significant for all 5 factors. These composite endophenotype factors will be used to enhance our neurobiological and genetic understanding of schizophrenia and its treatment as we move forward, and are related to the Biotype concept of psychosis. Larry Seidman was also an important contributor to the COGS mission as described below.

**Results:**

The COGS PsychChip GWAS of quantitative endophenotypes has now identified six regions of association with quantitative neurocognitive measures exceeding genome-wide significance (e.g. NRGL3-Abstaction and Mental Flexibility on the CNB). In addition, many associations between endophenotypes and specific loci exceed the suggestive threshold for further investigation.

**Discussion:**

These data will be presented implicating synaptic plasticity and other crucial CNS processes in endophenotype dysfunction in SZ. NB: COGS interrogates the genetic architecture of endophenotypes associated with SZ and its functional outcome, not schizophrenia per se. Still, there is much overlap between risk genes for SZ and neurocognitive endophenotypes. Also neurocognitive endophenotypes are endorsed by the FDA and MATRICS as treatment targets for schizophrenia itself. This allows for data-guided drug and sensory-cognitive remediation of neurocognitive deficits to improve functional outcome in schizophrenia. Using this genomic information to enhance precision based selection of treatment options now seems to be an exciting and viable new treatment pathway.

